# Endothelial Function in Children with Acute Lymphoblastic Leukemia (ALL) May Reflect the Clinical Outcome

**DOI:** 10.1155/2018/7918091

**Published:** 2018-11-11

**Authors:** Adrian Doroszko, Ewa Niedzielska, Maciej Jakubowski, Julita Porwolik, Aleksandra Turek-Jakubowska, Ewa Szahidewicz-Krupska, Bartosz Sieczkowski, Piotr Dobrowolski, Aneta Radziwon, Robert Skomro, Arkadiusz Derkacz, Grzegorz Mazur, Alicja Chybicka, Andrzej Szuba

**Affiliations:** ^1^Department of Internal Medicine, Hypertension and Clinical Oncology, Faculty of Medicine, Wroclaw Medical University, 213 Borowska St., Wroclaw, 50-556, Poland; ^2^Department of Pediatric Oncology, Hematology and Bone Marrow Transplantation, Wroclaw Medical University, 213 Borowska St., Wroclaw, 50-556, Poland; ^3^Clinic of Neurology, Regional Hospital, Rzeszów, Poland; ^4^Department of Congenital Heart Diseases, Institute of Cardiology, Warsaw, Poland; ^5^Glostrup Research Institute, Glostrup, Denmark; ^6^Division of Respiratory and Critical Care Medicine, Department of Medicine, University of Saskatchewan, Saskatoon, Canada; ^7^Division of Angiology, Faculty of Health Science, Wroclaw Medical University, 5 Bartla St., Wroclaw, 51-618, Poland

## Abstract

Endothelial dysfunction is a common feature of early complications of hemato-oncologic therapy. The aim of our study was to assess the profile of endothelial function at diagnosis time, then during initial treatment phase of acute lymphoblastic leukemia (ALL), and to verify the presence of its correlation with early clinical outcome (ECO). 28 ALL children and 18 healthy age-matched control ones were recruited. Study group was examined at baseline and at 33rd and 78th day of treatment. At each protocol step the endothelial function was assessed by measurement of sP-selectin (CD62-P), PAI-1(serpinE1), sE-selectin (CD62E), sICAM-1(sCD54), sVCAM-1(sCD106), and VEGF concentrations. Higher baseline sICAM-1 and sVCAM-1 levels and lower sP-selectin and VEGF were observed in children with ALL. sICAM-1, sVCAM-1, and sE-selectin levels were decreasing following the treatment with protocol I. Higher sE-selectin and lower baseline sICAM-1 levels were observed in children treated unsuccessfully. Lower PAI-1 levels were observed in children who survived. Higher baseline sE-selectin levels and lower sICAM-1 and VEGF were observed in children treated unsuccessfully. A decrease in sE-selectin and lower PAI-1 at the 78th day of therapy were associated with better ECO. High baseline VEGF and sE-selectin levels, significant increase in PAI-1, and low initial sICAM-1 levels are prognostics for poorer prognosis in the ALL children.

## 1. Introduction

Activation of vascular endothelium under pathological conditions is associated with an increased risk of death in numerous severe diseases [1–4]. Endothelial dysfunction (ED) is a common feature of many early complications of hemato-oncologic therapy which remain significant cause of morbidity and mortality despite the continuous optimization of treatment protocols [5]. Furthermore, their pathogenesis remains poorly understood.

Up to date, there are some established determinants of the endothelial cell injury development, but in a vast majority they are connected with chemotherapeutics (especially calcineurin inhibitors), methods used for conditioning (cytostatic, total body irradiation (TBI)), and certain aspects of the bone marrow transplant procedure itself [6]. However, direct risk stratification associated with the present treatment is difficult due to the complexity and multiplicity of interactions, which can in different ways and magnitude generate dysfunction and structural damage to the endothelium. In our previous publication we showed that endothelial dysfunction belongs to pathologies observed in childhood ALL prior to treatment and it may result from increased plasma concentration of ADMA (asymmetric dimethylarginine). Additionally, at 78th day of treatment we observed both improvement in endothelial function and reduction of plasma ADMA concentration. Interestingly, it was accompanied by reduction of plasma prostacyclin concentration [7]. Recognizing endothelial dysfunction as a multifactorial disease, we intended to broaden in this paper the spectrum of analyses.

Under physiological condition there is a continuous balance in an interplay between chemokines acting pro- and antiaggregatory (such as PAI-1, sE-selectin, sP-selectin) as well as pro- and anti-inflammatory (i.e., TxB2, 6-keto-PGF-1*ɑ*, sVCAM-1, sICAM-1) leading to the maintenance of vascular homeostasis and determining appropriate endothelial function [8]. Furthermore, the critical localization of endothelial cells between streaming blood and remaining components of vascular wall places them in a pathophysiological mainstream of cardiovascular consequences of hemato-oncological malignances.

ALL is an aggressive disease characterized by accumulation of immature malignant cells and the risk for relapse varies between patients and depends not only on genetic abnormalities, but also on growth factors action. An inverse relationship between blast proliferation and the magnitude of response to growth factors has been observed [9]. Furthermore, there are some reports stating that there are increased levels of soluble intercellular adhesion molecule-1 (sICAM-1) [10], soluble vascular cell adhesion molecule-1 (sVCAM-1), soluble E-selectin [11], thrombomodulin, and von Willebrand factor [12] in ALL children at the time of diagnosis, which indicates that ED may be present before the treatment begins.

To summarize, in this study we intended to clarify these relationships and verify the usefulness of analyzing selected markers of endothelial dysfunction for early risk stratification, as well as the ability to monitor the severity of complications. Hence, the aim of our observational study was to assess the profile of endothelial function at the diagnosis time as well as during initial phase of treatment and to verify the presence of its correlation with an early clinical outcome.

## 2. Material and Methods

All experiments were conducted and approved in accordance with the guidelines of the Bioethics Committee at Wroclaw Medical University and adhered to the principles of the Declaration of Helsinki and Title 45, US Code of Federal Regulations, Part 46, Protection of Human Subjects (revised November 13, 2001; effective December 13, 2001). All participants provided their informed consent which was followed by its written approval by a legal representative, as appropriate. The study and the written consent form had been approved by the Bioethics Committee at Wroclaw Medical University.

### 2.1. Patients and Controls

We have enrolled to our study 28 children with acute lymphoblastic leukemia and 18 healthy demographically matched children (Table 1).

**Figure 1:**
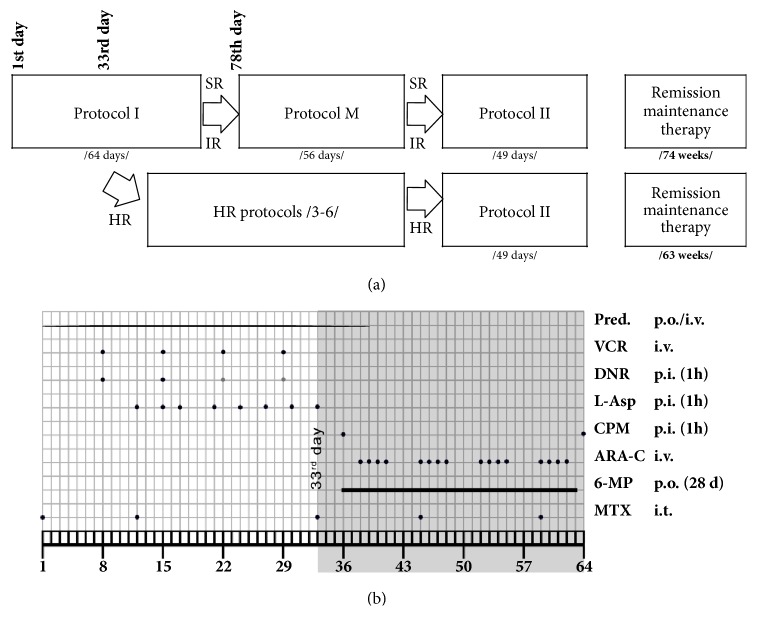
(a) Study design. (b) Treatment protocol. Protocol I in detail. Study covered the period of treatment common to all risk groups.* Abbreviations.* Pred, prednisone 60 mg/m^2^/d; VCR, vincristine 1.5 mg/m^2^/d; DNR, daunorubicin 30 mg/m^2^/d; L-Asp, L-Asparaginase 5000 U/m^2^/d; CPM, cyclophosphamide 1000 mg/m^2^/d; ARA-C, cytarabine 75 mg/m^2^/d; 6-MP, 6-mercaptopurine 60 mg/m^2^/dl; MTX, methotrexate; p.o., per os; p.i., per infusion; i.t., intrathecal; d 1, day 1; d 33, day 33; SR, standard risk; IR, intermediate risk; HR, high risk.

The study group included children with established diagnosis of acute lymphoblastic leukemia treated strictly according to the guidelines published in ALL-IC BMF 2002 protocol [13] (Figure 1(b)) in the Department of Pediatric Bone Marrow Transplantation, Oncology and Hematology, Wroclaw Medical University (Wroclaw, Poland). Children were examined at the baseline (day of diagnosis), on the 33rd and 78th day of therapy. At all three points, blood samples had been obtained and endothelial function was assessed (Figure 1(a)).

ALL children were categorized into three risk groups (standard /n=8/, intermediate /n=14/, and high risk /n=6/). Independently of this fact, during the first 78 days all of them were treated in the same way with protocol I. The only difference was a doubled dose of daunorubicin in intermediate and high risk groups. The protocol M was implemented for the low-to-intermediate risk children, whereas the HR protocol was used for children with high risk. This was followed in turn by protocol II used in all the ALL children independently on the baseline risk (Figure 1). Remission was observed in all patients at the 33rd day of treatment.

The control group (n=18) was formed by healthy children hospitalized at general pediatrics ward (Wroclaw Medical University, Wroclaw, Poland) due to disorders that do not affect endothelial function. Controls were examined only once, since no changes in the profile of endothelial function were suspected.

### 2.2. Biochemical Tests

Blood was collected using the Sarstedt S-Monovette® system (Sarstedt AG & Co., Nümbrecht, Germany). Serum (7.5 mL) and EDTA plasma (9.0 mL; 1.6 mg-EDTA/ml of blood) were separated, immediately centrifuged (1000 x g for 15 minutes at 4°C), and frozen at −20°C for evaluation markers of endothelial activation.

### 2.3. Markers of Endothelial Activation

Plasma concentrations of sP-selectin (CD62-P) and PAI-1 (serpin E1) and serum concentrations of sE-selectin (CD62E), sICAM-1 (sCD54), sVCAM-1 (sCD106), and VEGF were determined by a sandwich enzyme immunoassay technique, using commercial ELISA kits (R&D Systems Europe. Ltd., 19 Barton Lane Abingdon Science Park OX14 3NB, United Kingdom) according to the manufacturer's instructions, kits catalogue numbers: BBE6, DSE100, DSLE00, DCD540, DVC00, DVE00, respectively. The coefficient of variation (*CV*), intra-assay %CV, was calculated as the ratio of the pooled standard deviation from all samples (each was analyzed in triplicate) and the overall mean and then multiplied by 100. Inter-assay %CV refers to assay-to-assay consistency that was calculated using the pooled standard deviation divided by the overall mean of all duplicated samples and then multiplied by 100. The intra-assay and inter-assay %CVs were, respectively, 5.2% and 7.6% for sP-selectin, 6.1% and 7.2% for PAI-1, 7.6% and 9.7% for sE-selectin, 5.3% and 7.1% for sICAM-1, 3.7% and 7.6% for sVCAM-15.8, and 7.2% for VEGF.

### 2.4. Other Biochemical Analyses

Concentrations of serum creatinine, urea, fasting plasma glucose, Aspartate transaminase (AST), Alanine transaminase (ALT), lactate dehydrogenase (LDH), high sensitivity C-reactive protein (hsCRP), potassium, and uric acid were measured using standard commercial laboratory assays.

### 2.5. Statistical Analysis

Data is expressed as the mean ± SEM. The differences between two continuous parameters were assessed using a Mann-Whitney U-test or a Student's t-test, followed by a Shapiro-Wilk test and Levene's test as appropriate. For comparison of more than two groups, an ANOVA followed by Tukey's test or a Friedman ANOVA test (for nonparametric statistics) was performed. Correlations between continuous variables were calculated using Spearman test. All calculations were made using Statistica 10.0 StatSoft® and the graphical representation of the data was performed using GraphPad 5 Prism®_._

## 3. Results

### 3.1. General Characteristics of Groups

**Figure 2:**
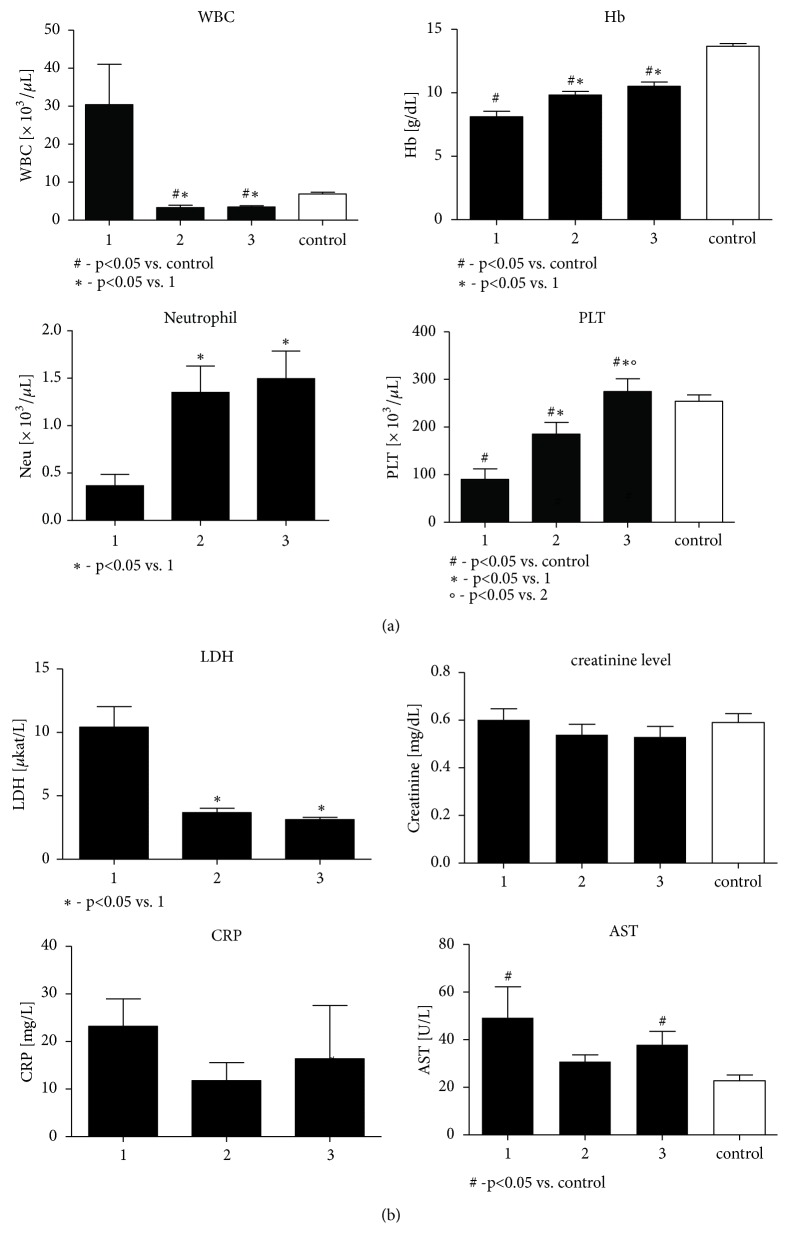
1. day, 33. day, M (the protocol M beginning day)—points of evaluation in the study group. (a) Complete peripheral blood cell counts in children with ALL at particular steps of the study protocol and in the control group. (b) Basic biochemical characteristics of children with ALL at particular steps of the study protocol and of the control group.

Both groups were similar regarding baseline demographic characteristics (Table 1). According to the expectations, there were significant differences related to the complete peripheral blood cell counts in children with ALL when compared to the control group (Figure 2(a)). Fasting glycemia was higher in ALL children, as compared to the control at each step of the study protocol. The LDH levels were higher at the onset of ALL management; however in the course of treatment they recovered to the physiological normal range. Basic biochemistry results are presented in Figure 2(b) and Table 1.

### 3.2. Endothelial Activation Markers

**Figure 3:**
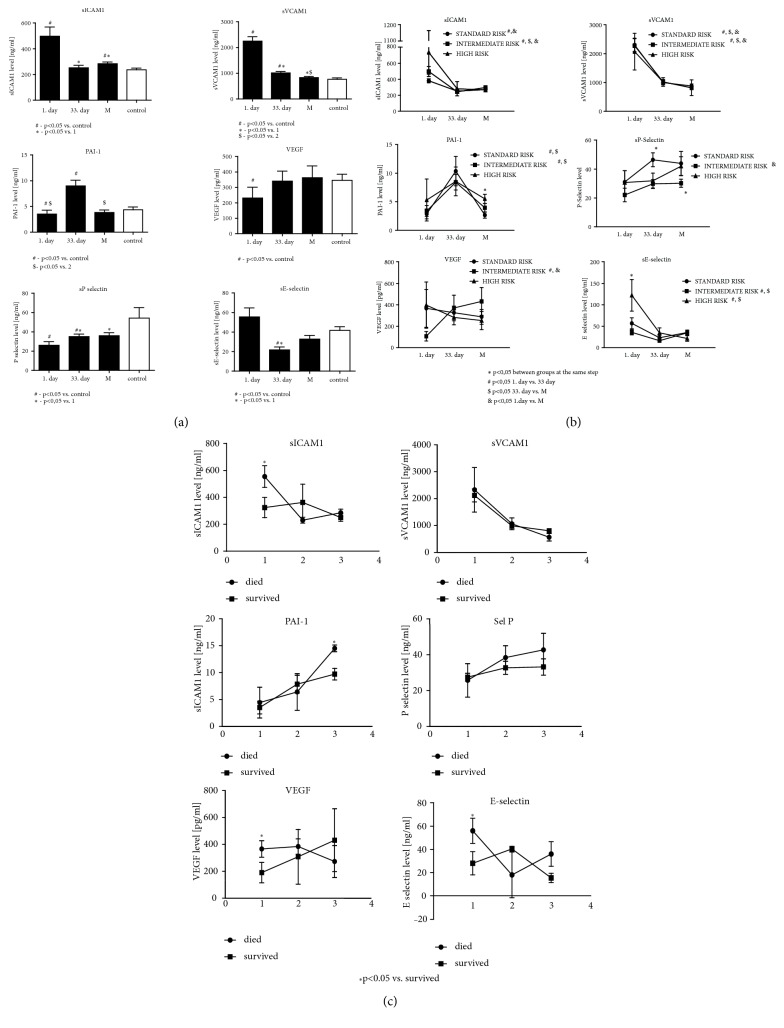
1. day, 33. day, M (the protocol M beginning day)—points of evaluation in the study group. (a) Endothelial activation and systemic inflammation markers in children with ALL at particular steps of the study protocol and in the control group. (b) Analysis of endothelial activation and systemic inflammation markers in children with ALL assigned to the subgroups separated according to the risk stratification. (c) Analysis of endothelial activation and systemic inflammation markers in children with ALL assigned to the subgroups separated according to the short-term survival.

Baseline sICAM-1 and sVCAM-1 levels, reflecting systemic inflammatory activation of endothelium, were significantly higher than those in control group. During treatment they significantly decreased, but still maintained higher levels compared to the control (Figure 3(a)). When analyzing the sICAM-1 and sVCAM-1 levels in the subgroups separated according to the risk, decreasing trends of the concentrations were observed only in the standard and intermediate risk groups (Figure 3(b)).

Baseline PAI-1 level in children with ALL was lower than that in controls. At 33rd day of observation the highest PAI-1 levels were observed compared to both control group and ALL children at other steps of the protocol (Figure 3(a)). These differences were significant in the whole study group, standard and intermediate risk group, but not in the high risk one. Furthermore, the high risk group was characterized by greater PAI-1 levels at the beginning of the protocol M (Figure 3(b)).

Analysis of the proaggregatory activation of endothelium, as assessed by the sE-selectin concentrations at the beginning and at the end of study time, did not differ significantly from control group. However, at 33rd day of treatment they were significantly lower when compared to both the control group and its baseline levels (Figure 3(a)). Additionally, baseline sE-selectin concentration in the high risk group was significantly higher as compared with the two remaining groups (Figure 3(b)). Significant decrease in the sE-selectin levels in the course of therapy was observed when compared both to the baseline values and to the control group.

Baseline VEGF and sP-selectin levels were significantly lower than values observed in further observation in whole study population and were also lower when compared to the control (Figure 3(a)). In ALL children, the sP-selectin concentrations at baseline were lower than during further treatment and also compared to control group. 33rd day levels (but not levels at the protocol M beginning) were significantly lower than ones in controls (Figure 3(a)).

### 3.3. Correlations with Cellular Lysis Markers

Statistically significant positive correlations between sVCAM-1 and AST as well as between sICAM-1 and uric acid levels were observed (Table 2).

### 3.4. Profile of Endothelial Function and a Short-Term Event-Free Survival (EFS)

Analysis of the subgroups separated according to the outcome (a short-term survival in a 12-month follow-up from the date of enrollment to the study) has shown that higher baseline sE-selectin levels as well as marked increase in PAI-1 levels during the treatment were characterized with poor prognosis. Similarly, higher baseline VEGF levels and sICAM-1 were associated with poorer EFS (Figure 3(c)).

## 4. Discussion

Our data broadens the scope of knowledge regarding pathophysiological abnormalities involved in endothelial dysfunction in ALL children. In this study we have demonstrated that proinflammatory and proaggregatory activation of endothelium is a common phenomenon in children with acute lymphoblastic leukemia. Moreover, serum cytokine and adhesion molecule profile in children with ALL may reflect important clinical characteristics related to both the risk profile and the outcome. Endothelial function in the study group at baseline was demonstrated to be partially impaired by endothelial inflammatory activation that accompanies the disease, which was reflected by elevated levels of sICAM-1 and sVCAM-1 [14]. Their concentrations are also positively correlated with cellular lysis markers (AST, uric acid) confirming thus that ED is a natural consequence of ALL. Cellular lysis resulting from increased cellular turnover in ALL seems thus to induce inflammation leading to development of endothelial dysfunction. In our previous work we showed that cellular lysis seems to cause also increased plasma concentration of ADMA, which is another established factor impairing endothelial function [7]. We verified that there is positive correlation between increase in concentration of ADMA and sVCAM-1 (r=0,61; p=0,01; Spearman's rank correlation coefficient; data not shown before).

In our study greater serum sICAM-1 levels, reflecting systemic inflammatory response, were associated with poor outcome and were noted in groups with greater than standard risk at baseline. This confirms the results presented in papers by Tacyildiz [10] and by Abdelrazik [15]. sICAM-1 plays a role in the immune response against tumor cells mediating the T cell cytotoxic response [16]. Aside from the fact that elevated levels of sICAM-1 represent an unfavorable prognostic marker in human neoplasias, sICAM-1 can be used to monitor the course of the disease [17] Similarly, increased serum levels of sVCAM-1 have been associated with tumor progression and metastatic potential in several solid malignancies. sVCAM-1 is normally expressed by bone marrow stromal cells, vascular endothelial cells, and follicular dendritic cells and its production is triggered by cytokines. In immunohistochemistry studies, VCAM-1 overexpression was found in biopsy material from acute leukemias [18]. Although the exact mechanism of release remains unknown, it is likely that sVCAM-1 is a product of enzymatic cleavage from endothelial or neoplastic cell surfaces induced by cytokines. Hence, determination of serum level of sICAM-1 and sVCAM-1 in ALL might represent an additional, but probably not independent, disease-associated marker for use in the clinical management of the children with acute lymphoblastic leukemia. Nonetheless, further studies are needed to establish if they could be used as a clinically relevant biomarkers in ALL management.

In this study we have shown for the first time the profile of changes in serum E-selection levels in pediatric ALL in relation to the risk group and outcome. Increased activation of endothelial cells at the onset of therapy (as reflected in sE-selectin concentrations) was associated in our study with worse prognosis and was present in children qualified to the high risk group at the beginning of therapeutic protocol. Other studies have shown similar effect of elevated serum E-selectin levels but either in other hematological malignancies [19] or in context of severe immunological complications of ALL [20].

Another marker of the ALL course considered in this paper is an endothelial plasminogen activator inhibitor (PAI-1). Plasminogen activator (PA) is implicated in solid tumor growth, invasion, and metastasis. However, little is known about its role in leukemia. Some studies have shown that PA and PAI-1 are synthesized by leukemic cells, thus pointing out that plasminogen activation may contribute to the invasive behavior of these cells [21]. In clinical studies, high levels of PAI-1, induced by corticosteroid treatment, lead to fibrinolysis suppression through inhibition of tissue plasminogen activator and thrombosis promotion[22]. As a result increased intraosseous venous pressure blocks blood flow to the femoral head and culminates in hypoxic bone death or osteonecrosis. It affects up to one-third of patients treated for ALL [23]. In our study, greater PAI-1 levels at the beginning of the protocol M were both associated with poorer prognosis (assessed by event-free survival /EFS/) and more predominant in children qualified to the high risk group at the beginning of therapeutic protocol. Hence, we postulate that the analysis of an increase in PAI-1 levels in children in the course of treatment could reflect increased risk for thrombotic complications. Nonetheless, verification of the usefulness of PAI-1 level in prospective follow-up of children with ALL during the therapy requires future large-number clinical trial.

Vascular Endothelial Growth Factor (VEGF) has been demonstrated to regulate the physiological and pathological angiogenesis. Its expression is associated with proliferation of neoplastic cells, their invasion and metastasis [24] VEGF may also play some role in pathogenesis of leukemia, since deregulation of VEGF expression and signaling pathways are observed in ALL [25]. Our data confirms studies by Kalra et al. [26] and Dincaslan et al. [27], demonstrating that untreated children with ALL have significantly lower VEGF concentration than controls and that, at the end of the induction therapy, VEGF increases to levels similar to controls [26, 27]. Hence, we confirm also the hypothesis that since VEGF is expressed in normal hematopoietic cells, the renewal of normal hematopoiesis in the course of therapy may explain the increase in VEGF levels and be a predictor of good response to the chemotherapy. This confirms also the data from the study by Avramis et. al stating that high VEGF levels in induction are a predictive marker for EFS [28].

As observed in our study proaggregatory and proinflammatory endothelial activation may lead in the long-term observation to the earlier onset of cardiovascular disorders in this subpopulation. Hence, we believe that the cardiovascular follow-up of the grown up childhood cancer survivors should be recommended.

## 5. Conclusions

Proinflammatory and proaggregatory activation of endothelium is a common phenomenon in children with acute lymphoblastic leukemia. High baseline serum levels of VEGF, high initial sICAM-1 and sE-selectin, and significant increase in PAI-1 levels are prognostics for poorer prognosis in a short-term observation in children treated for ALL. Evaluation of serum cytokine and adhesion molecule profile in children with ALL may reflect important clinical characteristics. However, further, larger clinical studies are needed in order to establish their exact usefulness in predicting the short- and long-term outcome in children with acute lymphoblastic leukemia.

## Figures and Tables

**Table 1:** Baseline characteristics of ALL children and control group.

Parameter	Study group	Control group			p
	(mean ± SEM)	(mean ± SEM)			
N	28	18			ns
Men (*n*)	12	6			ns
Age (*years*)	8.1 ± 1	11 ± 0.9			ns
ALT (U/L)	51.6 ± 32.3	17.7 ± 2.1			ns
AST (U/L)	49.2 ± 13	22.8 ± 2.4			0.02
Urea (mg/dL)	25.4 ± 1.6	21.3 ± 1.6			ns
Serum creatinine (mg/dL)	0.6 ± 0.0	0.6 ± 0.0			ns
Plasma glucose (mg/dL)	100 ± 5.9	80.4 ± 1.1			0.02

**Detailed baseline characteristics of the study group**					
	(mean ± SEM)				

CRP (mg/L)	23.2 ± 5.73				
Total bilirubin (mg/dL)	0.57 ± 0.11				
LDH (*µ*kat/L)	10.4 ± 1.6				
Total protein (g/dL)	6.8 ± 0.1				
Uric acid (mg/dL)	5.9 ± 0.5				
Sodium (mmol/L)	140 ± 0.4				
Potassium (mmol/L)	4.2 ± 0.1				

*Abbreviations.* AST: aspartate transaminase. ALT: alanine transaminase. hsCRP: C-reactive protein. LDH: lactate dehydrogenase. ns: not significant.

**Table 2:** Correlations between parameters assessed in the study group at baseline.

Parameters			Spearman's rank correlation coefficient	p
sVCAM-1	&	AST	0.51	0.01
sICAM-1	&	uric acid	0.44	0.03
sICAM-1	&	ADMA	0.61	0.01

*Abbreviations.* AST: aspartate transaminase, sVCAM-1: soluble vascular cell adhesion molecule 1, sICAM-1: soluble intercellular adhesion molecule 1.

## Data Availability

All the original data of the study used to support the findings are included within the article. The raw data used to support the findings of this study are restricted by the Bioethics Committee at Wroclaw Medical University in order to protect the patients' privacy.
